# An increase in physical activity after colorectal cancer surgery is associated with improved recovery of physical functioning: a prospective cohort study

**DOI:** 10.1186/s12885-017-3066-2

**Published:** 2017-01-25

**Authors:** Moniek van Zutphen, Renate M. Winkels, Fränzel J. B. van Duijnhoven, Suzanne A. van Harten-Gerritsen, Dieuwertje E. G. Kok, Peter van Duijvendijk, Henk K. van Halteren, Bibi M. E. Hansson, Flip M. Kruyt, Ernst J. Spillenaar Bilgen, Johannes H. W. de Wilt, Jaap J. Dronkers, Ellen Kampman

**Affiliations:** 1 Division of Human Nutrition, Wageningen University & Research, P.O Box 17, 6700 AA Wageningen, The Netherlands; 2 Department of surgery, Gelre Hospital, Apeldoorn, The Netherlands; 3 Department of Internal Medicine, Admiraal de Ruyter Hospital, Goes/Vlissingen, The Netherlands; 4 Department of Surgery, Canisius Wilhelmina Hospital, Nijmegen, The Netherlands; 5 Department of Surgery, Gelderse Vallei Hospital, Ede, The Netherlands; 6 Department of Surgery, Rijnstate Hospital, Arnhem, The Netherlands; 7 Department of Surgery, Radboud University Nijmegen Medical Centre, Nijmegen, The Netherlands; 8 Department of Physical Therapy, Gelderse Vallei Hospital, Ede, The Netherlands

**Keywords:** Recovery of function, Colorectal surgery, Colorectal cancer, Physical activity, Rehabilitation, Epidemiology

## Abstract

**Background:**

The influence of physical activity on patient-reported recovery of physical functioning after colorectal cancer (CRC) surgery is unknown. Therefore, we studied recovery of physical functioning after hospital discharge by (a) a relative increase in physical activity level and (b) absolute activity levels before and after surgery.

**Methods:**

We included 327 incident CRC patients (stages I–III) from a prospective observational study. Patients completed questionnaires that assessed physical functioning and moderate-to-vigorous physical activity shortly after diagnosis and 6 months later. Cox regression models were used to calculate prevalence ratios (PRs) of no recovery of physical functioning. All PRs were adjusted for age, sex, physical functioning before surgery, stage of disease, ostomy and body mass index.

**Results:**

At 6 months post-diagnosis 54% of CRC patients had not recovered to pre-operative physical functioning. Patients who increased their activity by at least 60 min/week were 43% more likely to recover physical function (adjusted PR 0.57 95%CI 0.39–0.82), compared with those with stable activity levels. Higher post-surgery levels of physical activity were also positively associated with recovery (P for trend = 0.01). In contrast, activity level before surgery was not associated with recovery (P for trend = 0.24).

**Conclusions:**

At 6 month post-diagnosis, about half of CRC patients had not recovered to preoperative functioning. An increase in moderate-to-vigorous physical activity after CRC surgery was associated with enhanced recovery of physical functioning. This benefit was seen regardless of physical activity level before surgery. These associations provide evidence to further explore connections between physical activity and recovery from CRC surgery after discharge from the hospital.

## Background

Surgery for colorectal cancer (CRC) is followed by a period of recovery which begins in hospital and continues after discharge [1, 2]. Postoperative recovery is a complex process encompassing physical, psychological, and social elements [1]. Clinicians have mainly focused their interest on assessing the in-hospital phases of recovery [1–3], but from a patient’s perspective recovery is only complete when the patient returns to normal function in day-to-day life [1, 2, 4]. Therefore, recovery might be best estimated with measures of functional status [1].

Functional status is often evaluated with patient-reported outcomes, for example with physical functioning [5, 6] or activities of daily living [7]. Low physical functioning is associated with disability and a loss of independence [8]. Following a rapid decline after CRC surgery [1, 9, 10], patient physical function scores return to pre-operative values [9, 10]. However, not all individual CRC patients recover to their pre-operative level of physical functioning. In a study among patients over 60 years of age undergoing major abdominal surgery for mixed reasons, less than 50% of patients recovered to baseline levels of functional status at 6 months after surgery [11]. Furthermore, 10% of patients were still unable to perform basic activities of daily living [11]. Recovery depends on clinical factors such as location of the tumor, presence of an ostomy, and patient characteristics (age and physical functioning before surgery) [12, 13].

Apart from patient and clinical factors, recovery of physical functioning could also be influenced by physical activity. Several studies consistently indicate that physically active older adults [14, 15] and physically active CRC survivors [6, 16–21] have higher physical functioning. The influence of physical activity on recovery of physical functioning after CRC surgery is unknown. Therefore, the aims of the present study are first to assess the proportion of CRC patients without patient-reported recovery of physical functioning at 6 months post-diagnosis. Second, we examine the association between patient-reported recovery of physical functioning and (a) an increase in moderate-to-vigorous physical activity from pre-to-post surgery and (b) absolute activity levels before and after surgery.

## Methods

### Study population

This study is embedded in the COLON-study [22]. In this prospective cohort study, data were collected from newly diagnosed CRC patients in any stage of the disease. Patients were excluded when they had a history of colorectal cancer or (partial) bowel resection, chronic inflammatory bowel disease, a known hereditary colorectal cancer syndrome, dementia or another mental condition, or were non-Dutch speaking. Eligible participants were invited by hospital staff to participate in the study during a routine clinical visit before scheduled surgery. Response rates varied from 35 to 70% in the four hospitals that reported non-responders; overall response rate was estimated to be 50%. Approval for the study was obtained from the Committee on Research involving Human Subjects, region Arnhem-Nijmegen (The Netherlands) and all participants provided written informed consent.

Participants were asked to fill out several mailed questionnaires shortly after diagnosis, but before start of clinical treatment, and 6 months later. Individuals in the current analysis included all COLON-study participants that were recruited between August 2010 and November 2013. Follow-up data collection was completed in May 2014.

### Physical functioning

Physical functioning was assessed using the validated European Organization for Research and Treatment of Cancer quality-of-life questionnaire (EORTC QLQ-C30), translated in Dutch [23]. The physical functioning scale contained five questions (trouble with strenuous activities / long walk / short walk / need to stay in bed or chair during the day / basic activities of daily living). The answers ranged from ‘not at all’ to ‘very much’. A summary score that ranged from 0 (worst) to 100 (best) was calculated according to the EORTC scoring manual [24]. At 6 months post-diagnosis patients were considered to be either recovered or not recovered. No recovery of physical functioning was predefined as a physical functioning score at 6 months post-diagnosis that was at least five points lower than before surgery. This decrease is considered a clinically relevant change [25].

### Physical activity

Physical activity was assessed using the validated Short QUestionnaire to ASsess Health enhancing physical activity (SQUASH) [26–28]. Participants were asked to report their average time (days per week, hours and minutes per day) spent in walking, cycling, gardening, odd-jobs, sports, household activities and work. Based on the self-reported intensity level of each activity a metabolic equivalent (MET) value was assigned [29]. We used 3.3 MET as the lower cut-off for moderate activity [15]. However, in accordance with the SQUASH manual and the Dutch physical activity guideline, 4.0 MET was used as a cut-off value for those aged <55y. The change in physical activity from pre-to-post surgery was classified into three pre-defined groups (stable, increase and decrease). When pre-to-post surgery moderate-to-vigorous physical activity changed less than 60 min/week, this was considered a stable activity level; otherwise it was classified as a decrease or increase in activity. CRC surgery might result in a prolonged low physical functioning and therefore a reduced ability to be physically active. A decreased post-operative physical activity level might thus be the result of not being recovered. Therefore, we made the a priori decision to focus our analysis on the group that had the ability to be active at pre-surgery level six months after diagnosis.

### Covariates

Socio-demographic characteristics, smoking, body mass index (BMI) and presence of comorbidities were assessed with a self-administered questionnaire shortly after diagnosis. Clinical characteristics (such as tumor location, stage of disease and treatment) were retrieved from medical record abstraction.

### Statistical analyses

Descriptive statistics were used to assess the proportion of CRC patients not recovered at 6 months post-diagnosis and to describe participant characteristics by recovery of physical functioning. Cox regression models (with robust error variance and constant risk period assigned to all participants) were used to calculate adjusted prevalence ratios (PRs) of no recovery of physical functioning at 6 months post-diagnosis. This method was chosen instead of logistic regression, because it is a better alternative for the analysis of binary outcomes [30]. A PR > 1.0 means that the proportion of people without recovery is greater in those with the exposure. A PR < 1.0 means there is a lower prevalence of people without recovery; in other words, more people with the exposure of interest are recovered when the PR < 1.0. The primary exposure of interest was an increase in physical activity from pre-to-post surgery. In addition, we examined the absolute level of physical activity before and after surgery in relation to recovery. Next, we stratified our main analysis on pre-surgery physical activity level, to explore if the magnitude of benefit was dependent on the starting level of physical activity. Age (years), sex, and physical functioning before surgery (score) were predefined covariates. Furthermore, stage of disease (I, II, and III), ostomy (yes, no), and BMI (kg/m^2^) were covariates in all models because they yielded a >10% change in the PR estimate. In addition to the main covariates described above, other potential confounders were evaluated for inclusion in the Cox regression models. However, none of the variables tested [living with a partner (yes, no), smoker before surgery (yes, no), cancer site (colon, rectum), neo-adjuvant therapy (yes, no), adjuvant chemotherapy (yes, no), ostomy reversal (yes, no), length of hospital stay >10 days (yes, no), and having one or more comorbidity (yes, no)] yielded an important change (<10%) in the PR estimate and were therefore not included. The P-value for the linear trend test across categories of physical activity was calculated by using the median value of each category as a continuous variable. All analyses were performed using SAS version 9.3 (SAS Institute Inc., Cary, NC).

## Results

### Participant characteristics

A total of 515 CRC patients were included in the COLON study. Patients were excluded from analysis when they had stage IV disease or an unknown disease stage (*n* = 63), did not undergo tumor resection (*n* = 7), had long course neo-adjuvant therapy (*n* = 45) or when post-surgery physical functioning was assessed within 8 weeks after tumor resection (*n* = 2). Furthermore, 71 patients were excluded from analyses since they did not provide any information on physical activity and/or physical functioning before surgery (*n* = 31) or 6 months post-diagnosis (*n* = 40). Therefore, a total of 327 participants were available for analyses.

At 6 months post-diagnosis (164 ± 25 days after tumor resection) 54% (*n* = 178) of CRC patients had not recovered to pre-operative physical functioning. Socio-demographic characteristics such as age, sex and education level were similar between the two groups (Table 1). Patients who had not recovered were more often smokers and had a BMI ≥ 30 kg/m^2^ compared with patients who had recovered. Furthermore, we observed that patients who had not recovered were more often rectal cancer patients and received additional treatment following surgery compared to patients who had recovered.

**Table 1:** Characteristics of colorectal cancer patients, overall and by patient-reported recovery of physical functioning at six months after surgery

	Total	Recovery of physical functioning	
		Yes	No
		46%	54%
	(*n* = 327)	(*n* = 149)	(*n* = 178)
Socio-demographic characteristics^a^			
Age (y)^b^	65 ± 10	66 ± 9	65 ± 10
Male	198 (61%)	87 (58%)	111 (62%)
Education level			
Low	155 (47%)	69 (46%)	86 (48%)
Middle	66 (20%)	31 (21%)	35 (20%)
High	106 (32%)	49 (33%)	57 (32%)
Living with partner	263 (80%)	119 (80%)	144 (81%)
Lifestyle characteristics			
Smoking status			
Never	99 (30%)	50 (34%)	49 (28%)
Former	188 (58%)	85 (58%)	103 (58%)
Current smoker before surgery	38 (12%)	12 (8%)	26 (15%)
Body mass index before surgery (kg/m^2^)			
< 20	13 (4%)	9 (6%)	4 (2%)
≥ 20–25	134 (41%)	63 (42%)	71 (40%)
≥ 25–30	141 (43%)	68 (46%)	73 (41%)
≥ 30	39 (12%)	9 (6%)	30 (17%)
≥ 150 min/week physical activity before surgery	281 (86%)	126 (85%)	155 (87%)
Physical activity before surgery (h/week)	9.0 (4.5–17.8)	8.5 (4.0–17.8)	9.8 (4.9–17.3)
Physical activity at six months post-diagnosis (h/week)	6.0 (2.0–11.5)	8.0 (4.0–14.1)	4.1 (0.8–8.3)
Physical activity difference (h/week)	−2.5 (-8.0–0.7)	−1.0 (-5.0–3.0)	−4.0 (-12.0--0.3)
Increase of ≥60 min/week of physical activity	81 (25%)	55 (37%)	26 (15%)
Clinical characteristics			
Colon cancer	233 (71%)	116 (78%)	117 (66%)
Rectal cancer	92 (28%)	32 (21%)	60 (34%)
Disease stage (pTNM)			
Stage I	96 (29%)	41 (28%)	55 (31%)
Stage II	112 (34%)	70 (47%)	42 (24%)
Stage III	119 (36%)	38 (26%)	81 (46%)
Neo-adjuvant therapy	73 (22%)	25 (17%)	48 (27%)
Adjuvant chemotherapy	91 (28%)	28 (19%)	63 (35%)
Ostomy	103 (32%)	35 (23%)	68 (38%)
Ostomy reversal	32 (10%)	14 (9%)	18 (10%)
Length of hospital stay > 10 days	82 (25%)	35 (23%)	47 (27%)
Days after surgery^b^	164 ± 25	167 ± 24	162 ± 25
Health status characteristics			
Comorbidity before surgery^c^	142 (43%)	57 (40%)	85 (60%)
Physical functioning before surgery	93.3 (86.7–100)	93.3 (80.0–100)	93.3 (86.7–100)
Physical functioning at six months post-diagnosis	86.7 (73.3–93.3)	93.3 (86.7–100)	73.3 (60.0–86.7)
Change in physical functioning	−6.7 (-13.3–0.0)	0.0 (0.0–6.7)	−13.3 (-26.7--6.7)

^a^All data are presented as *n* (%) or median (25^th^, 75^th^ percentile), unless otherwise indicated

^b^mean ± SD

^c^One or more of the following comorbidities: diabetes mellitus, chronic respiratory disease, and cardiovascular disease (excluding determinants of cardiovascular disease like high blood pressure)

Participants who did not provide information on physical activity and/or physical functioning (*n* = 71) were on average slightly older (69 y vs 65 y), female (51% vs 39%), rectal cancer patients (34% vs 28%), and of more advanced disease stage (stage III disease (44% vs 36%)), than the included subjects (*n* = 327).

### Increase in physical activity after surgery

**Fig. 1:**
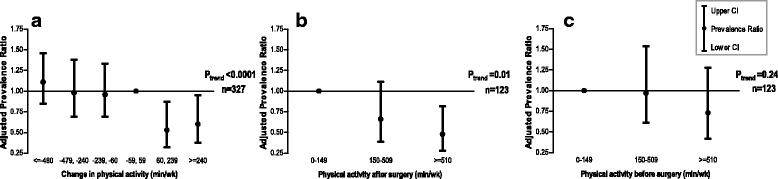
Prevalence ratio and 95% confidence intervals (CI) of the association between no recovery of physical functioning at 6 months post colorectal cancer diagnosis and **a** *change* in moderate-to-vigorous physical activity from pre-to-post surgery (*n* = 327), *n* = 87, 47, 70, 42, 41, 40 patients; or **b** absolute level of moderate-to-vigorous physical activity *after* surgery among the subset of patients that either increased their activity level or had a stable activity level from pre-to-post surgery (*n* = 123), *n* = 21, 41, 61 patients; or **c** absolute level of moderate-to-vigorous physical activity *before* surgery among the subset of patients that either increased their activity level or had a stable activity level from pre-to-post surgery (*n* = 123), *n* = 39, 47, 37 patients. Models adjusted for age, sex, physical functioning before surgery, stage of disease, ostomy, and body mass index

About 25% (*n* = 81) of patients were able to increase their level of physical activity from diagnosis to 6 months post-diagnosis. Those patients were 43% more likely to be recovered (adjusted PR 0.57; 95%CI 0.39–0.82) compared with patients who had a stable activity level (*n* = 42) (Table 2). When the increase in physical activity was split into two groups, both an increase of 60–240 min/week (adjusted PR 0.53; 95%CI 0.32–0.87) and an increase of ≥240 min/week (adjusted PR 0.60; 95%CI 0.38–0.95) showed similar associations with recovery (Fig. 1a).

**Table 2:** The association between no recovery of physical functioning after CRC surgery and stable or increased activity from pre-to-post surgery stratified by activity level before surgery

Moderate-to-vigorous activity level	No. events/ at risk	Adjusted PR (95% CI)	Adjusted PR (95% CI)
Stable activity	25/42	1.00	
Inactive^a^ with stable activity	12/20		1.00
Active^b^ with stable activity	13/22		0.91 (0.65–1.26)
Increased activity	26/81	0.57 (0.39–0.82)	
Inactive with increasing activity	6/19		0.53 (0.29–0.97)
Active with increasing activity	20/62		0.55 (0.39–0.78)

*Adjusted for age, sex, physical functioning before surgery, stage of disease, ostomy, and body mass index*

^a^Inactive is defined as a pre-surgery activity level <150 min/week

^b^Active is defined as a pre-surgery activity level >150 min/week

A sensitivity analyses was conducted whereby we repeated our analysis in the subsample of patients treated with surgery only (*n* = 168). This sensitivity analyses showed that patients who increased their activity level were 50% more likely to be recovered (adjusted PR 0.50; 95%CI 0.24–1.01) compared with patients who had a stable activity level.

### Physical activity after surgery

Higher post-surgery physical activity was positively associated with recovery among the subset of patients that either increased their activity level or had a stable activity level from pre-to-post diagnosis (*P* for trend = 0.01; Fig. 1b). Compared with patients who reported no moderate-to-vigorous activity per week, those reporting 510 or more minutes per week (8.5 h/week) were 52% more often recovered to their pre-operative level of physical functioning (adjusted PR 0.48; 95%CI 0.28–0.82).

### Physical activity before surgery

Pre-surgery physical activity was not associated with recovery of physical functioning among the subset of patients that either increased their activity level or had a stable activity level from pre-to-post diagnosis (*P* for trend = 0.24; Fig. 1c). Also within the total group of patients (*n* = 327) there was no association between physical activity level before surgery and recovery (*P* for trend = 0.55; results not shown).

### Increase in physical activity stratified by physical activity before surgery

We further subdivided patient groups of stable activity and increased activity, to assess whether the magnitude of benefit was dependent on physical activity level before surgery. For patients with stable activity, we divided participants into those engaging in <150 min/week (inactive with stable activity) and ≥150 min/week (active with stable activity). For patients with increased activity, we also defined two groups based on their pre-surgery activity level with a cut-off value of 150 min/week (inactive with increased activity and active with increased activity) (Table 2). Both groups of patients who increased their activity (irrespective of pre-surgery activity) were 45% more likely to be recovered to their pre-operative physical functioning (Table 2) compared to patients that were inactive before surgery and remained inactive. In contrast, patients who were active before surgery with stable activity after surgery were not more often recovered (adjusted PR 0.91; 95%CI 0.65–1.26).

## Discussion

The present study found that at 6 months post-diagnosis about half of CRC patients had not recovered to their pre-operative physical functioning. CRC patients who increased their activity from their levels before surgery were significantly more likely to be recovered compared to patients who had a stable activity level. Furthermore, patients who were physically active after CRC surgery were more likely to recover their physical functioning. In contrast, level of activity before surgery was not associated with recovery of physical functioning.

Few studies have assessed the association between physical activity and recovery of physical functioning after colorectal cancer surgery. Since recovery is defined as return to baseline function, quantification of recovery requires measurement both at baseline and after discharge from the hospital. Those data are not commonly reported. Several studies assessed in-hospital recovery [3], return to work [31], or assessed physical functioning only after surgery [9, 10, 32, 33]. We found that 54% of CRC patients had not recovered their pre-surgery physical functioning at 6-months post-diagnosis. Along with a previous study [11], these data suggest that a substantial proportion of patients have not recovered to preoperative functioning by 5 to 6 months post-surgery.

The main finding in the present study was that CRC patients who increased their physical activity levels above baseline levels were more often recovered from surgery. The magnitude of benefit of increasing activity was similar in patients who had either a high or moderate increase in activity and was independent of pre-surgery physical activity level. Our analyses also demonstrate that CRC patients who were consistently active (at least 150 min/week), but did not increase their activity, did not experience improved recovery. These results are in line with a previous study among cancer survivors, which concluded that it was the change in physical activity since cancer diagnosis that was associated with current physical functioning, rather than the absolute amount of physical activity [19]. However, a possible explanation for this finding is that an increase in physical activity level might be needed in order to regain muscle mass, aerobic capacity, and coordination [34]. Nonetheless, because this is the first study that assessed the impact of absolute levels and relative increases in activity on recovery after CRC surgery, these findings need to be confirmed. Future studies should preferably include multiple assessments of physical activity and physical functioning after surgery to better follow the recovery trajectory.

Furthermore, our results showed that pre-surgery activity was not associated with recovery. Several other studies have examined the effect of pre-surgery activity on recovery of physical functioning among CRC patients. In contrast to our result, one study concluded that a higher pre-operative physical activity level was associated with a faster self-reported recovery after surgery [35]. However, that study measured recovery at 3 and 6 weeks after surgery and only used the single question “to what extent do you feel physically recovered?” to measure recovery among 115 CRC patients. Our results are in line with a recent systematic review that concluded there is no evidence that pre-operative physical activity improves post-operative outcomes such as recovery in CRC patients [36].

The current study has some limitations that need to be taken into consideration when interpreting the results. First, our measurements were taken at six months post-diagnosis and not at six months post-surgery. However, the number of days since surgery was similar for those patients that did recover versus patients that did not recover at six months post-diagnosis. Furthermore, our results did not seem to be influenced by additional cancer treatment. In sensitivity analyses, in which we included patients treated with only surgical resection, we found a similar association between an increase in physical activity and recovery as in the total study population.

Another limitation is that recovery of physical functioning was measured using questionnaires based on self-report. Generally, the ceiling effect of the physical functioning scale is considered a limitation [37]. Many patients score the maximum of 100 on physical functioning before surgery. As a consequence, patients with the highest possible score cannot be distinguished from each other, while differences in physical functioning are present. Therefore, patients who score the maximum both before and months after surgery (*n* = 65, 20%) could still have experienced an overall decline in physical functioning, although we were not able to measure this decline. However, for this study we focused on a clinically relevant decline in physical functioning that resulted in a deterioration of the ability to cope independently [25], i.e. patients were considered not recovered from surgery. Ideally, both objective and self-reported measures should have been included to fully capture multiple domains of physical functioning. In a study among older patients undergoing major abdominal surgery, the proportion not recovered indeed varied across different measures [11]. In that study the proportion of patients without recovery was consistently greater with performance-based instruments than with self-reported measures of physical functioning [11]. We found that about half of patients were not recovered to their pre-surgery capacity to perform physical and daily routine activities. We do not expect that more patients would be considered to be recovered if we would have used objective measures of physical functioning.

Physical activity level was measured with self-reported questionnaires. Objective measures, such as accelerometers, are complementary to, rather than a replacement for, self-reported methods in epidemiologic studies. Accelerometers capture short-term measures of physical activity, while questionnaire are designed to give a representative view of habitual long-term physical activity. Physical activity levels of patients around the time of diagnosis may deviate significantly from their regular physical activity behaviour, e.g. because of frequent visits to the hospital. Therefore, accelerometers may be inappropriate to capture habitual physical activity before treatment, while questionnaires are.

Lastly, the response rate of 50% and missing data of some patients on exposure and/or outcome may limit the generalizability of our results. In addition, our study population was quite active; 86% of patients were active at or over the recommended 150 min/week. This is slightly higher than the general Dutch population aged 55+, in which 72% meets the physical activity guideline. However, this activity level was similar to the 91% adherence to the physical activity guideline that was found in another study among Dutch CRC survivors [38]. In contrast, the proportion of CRC patients meeting the activity recommendation in North-America and Australia are generally much lower [16, 18]. The high level of physical activity in our study population might limit the generalizability of our results to other populations of CRC patients. However, our results suggest that the benefit of an increase in physical activity is independent from the pre-surgery level of activity (<150 min/week vs. ≥150 min/week).

This study has several strengths. First, we were able to adjust for many covariates that could potentially confound our associations. Although no data was available about complications that occurred, length of hospital stay was used as an indicator of major complications after surgery. Second, the COLON study provided a unique opportunity to explore recovery after CRC surgery, since we measured physical functioning both before surgery and after discharge from the hospital. Third, we compared CRC patients who increased their activity levels after surgery with patients who had a stable activity level. No comparison was made with regard to patients who decreased their activity levels after CRC surgery, since CRC surgery might result in a prolonged low physical functioning and therefore a reduced ability to be physically active.

## Conclusions

Our results suggest that an increase in moderate-to-vigorous physical activity after CRC surgery is associated with enhanced recovery of physical functioning, independent of physical activity level before surgery. This benefit was seen regardless of age, stage of disease, BMI, or physical functioning before surgery. Furthermore, our results suggest that pre-surgery activity is not associated with recovery. The design of this study precludes any causal inference. The effects of pre-operative and post-operative physical activity on recovery should be further studied. Future prospective studies that investigate functional recovery are needed and should include more time points during follow-up to better follow the recovery trajectory. Moreover, randomized trials are needed to study if pre-operative and/or post-operative physical activity programs will enhance recovery. Randomized trials that examine the effects of post-operative physical activity programs should include pre-operative measures of both physical activity and functional status to be able to test the level of physical activity needed to enhance recovery.

## Abbreviations

BMI: Body mass index
CRC: Colorectal cancer
EORTC QLQ-C30: European Organisation for Research and Treatment of Cancer quality-of-life questionnaire
MET: Metabolic equivalent
PR: Prevalence ratio
SQUASH: Short QUestionnaire to ASsess Health enhancing physical activity.
